# Effects of skin‐to‐skin contact on full‐term infants’ stress reactivity and quality of mother–infant interactions

**DOI:** 10.1002/dev.22308

**Published:** 2022-07-17

**Authors:** Nicole Rheinheimer, Roseriet Beijers, Kelly H. M. Cooijmans, Bonnie E. Brett, Carolina de Weerth

**Affiliations:** ^1^ Department of Cognitive Neuroscience Donders Institute for Brain, Cognition and Behaviour, Radboud University Medical Center Nijmegen the Netherlands; ^2^ Behavioural Science Institute, Radboud University Nijmegen the Netherlands

**Keywords:** mother–infant interaction, skin‐to‐skin contact, stress regulation, term birth

## Abstract

Skin‐to‐skin contact (SSC) between mothers and their infants has beneficial effects in both preterm and full‐term infants. Underlying mechanisms are largely unknown. This randomized controlled trial assessed whether daily SSC in full‐term mother–infant dyads: (1) decreases infants’ cortisol and behavioral reactivity to a mild naturalistic stressor, and (2) facilitates interaction quality between infants and mothers (i.e., improved maternal caregiving behavior and mother–infant adrenocortical synchrony). Pregnant Dutch women (*N* = 116) were recruited and randomly allocated to an SSC or care‐as‐usual condition. The SSC condition performed 1 h of SSC daily, from birth until postnatal week 5. In week 5, mothers bathed the infant (known mild stressor). Infant and maternal cortisol was sampled at baseline, 25 and 40 min after bathing, and infant and maternal behavior was rated. Results did not indicate effects of SSC on infant behavioral and cortisol reactivity to the bathing session. Similarly, no effect of SSC was found on maternal caregiving behavior and mother–infant adrenocortical synchrony. In conclusion, the findings provide no evidence that daily mother–infant SSC is associated with full‐term infants’ behavioral and adrenocortical stress reactivity or mother–infant interaction quality. Future studies should replicate these findings and unveil other potential mechanisms underlying beneficial effects of SSC.

## 1 INTRODUCTION

Skin‐to‐skin contact (SSC) is very beneficial for young infants. In both preterm and full‐term infants, SSC has, for instance, been shown to improve health outcomes, facilitate sleep, and decrease crying behavior (Campbell‐Yeo et al., 2015; Moore et al., 2016; Norholt, 2020). Nevertheless, there is a lack of randomized controlled trials (RCT) on the effects of SSC, and it is unclear whether SSC in the hours immediately after birth is more beneficial than delayed SSC procedures (Moore et al., 2016). Moreover, underlying working mechanisms of SSC are widely unknown. Researchers hypothesize that the close contact enables mothers to provide sensory cues (e.g. touch, odor, vocalizations), which facilitate the development of self‐regulation of the infant (Feldman, 2012b; Feldman et al., 2002; Norholt, 2020). Accordingly, studies on preterm infants found positive effects of daily SSC on infants’ biological and behavioral reaction to stress (Feldman et al., 2014; Ionio et al., 2021; Mörelius et al., 2015). Furthermore, research in preterm infants also indicates that daily SSC improves the mother–infant interaction quality, in terms of maternal caregiving behavior toward the infant, and mother–infant synchronization of biological processes (Feldman et al., 2014; Mörelius et al., 2015). In this RCT, we assess whether daily SSC affects full‐term infants’ stress reactivity, as well as the quality of the mother–infant interaction.

When exposed to stressful situations, the hypothalamic–pituitary–adrenal (HPA) axis produces the hormone cortisol. Although this HPA axis reaction enables infants to cope with stressful situations, repeated elevations of the hormone cortisol can have a negative impact on physiological and mental health (Nelson et al., 2011; Radley et al., 2015). Human infants are born with an immature ability to regulate their biological and behavioral stress reactions (Schore, 2001). Hence, infants highly depend on external regulation, provided through interactive cues during close proximity with their caregiver (Hofer, 1987; Hostinar et al., 2014; Loman et al., 2010; McKenna & Mosko, 1994). During SSC, the infant, wearing only a diaper, is placed on the mother's bare chest (World Health Organization, 2003). This full‐body contact allows mothers to provide infants with essential regulatory cues, such as touch, warmth, and vocalizations (Feldman et al., 2014; Ionio et al., 2021). Accordingly, research indicates that a single episode of SSC significantly decreases baseline cortisol levels in full‐term infants (Beijers et al., 2016), and when performed prior to an injection stressor, SSC decreases infants’ crying response (Gray et al., 2000; Johnston et al., 2014).

The ability to regulate distress, including the functioning of the HPA axis, matures throughout infancy and is sensitive to environmental circumstances, such as continuous maternal proximity (Gunnar et al., 2009; Herman et al., 2016; Jansen et al., 2010b). According to Feldman's biobehavioral theory on parent–infant interactions, repeated mother–infant contact and the resulting exchange of biobehavioral cues in the first postnatal months, facilitate infants’ maturation of their ability to regulate autonomous stress reactions (Feldman, 2012a). In line with this, an RCT on preterm infants demonstrated that performing SSC daily in the first postnatal weeks, as compared with care‐as‐usual (CAU), decreased infants’ cortisol reactions to a stressor at one month of age (Mörelius et al., 2015). A study on full‐term infants also reported that infants who had received daily SSC for the first 6 postnatal weeks showed decreased cortisol reactivity to a stressor (Hardin et al., 2020). However, previous findings were not based on a randomized sample, and carried out statistical analyses on a small number of dyads who had adhered with the SSC intervention protocol, excluding infants of noncompliant mothers.

Apart from affecting physiological reactivity, daily SSC might also affect infants’ behavior during distress. Through repeated face‐to‐face interactions during SSC, infants are suggested to become familiarized with maternal cues, and hence learn to rely more on their mother when confronted by a challenging situation (Feldman et al., 2014; Tessier et al., 1998). Accordingly, studies on preterm infants report that infants who received repeated SSC in the first postnatal weeks, compared with CAU, showed increased responsivity to maternal cues, less gaze aversions, and decreased negative emotionality when exposed to a stressor (Chiu & Anderson, 2009; Feldman et al., 2002). Furthermore, Neu and Robinson (2010) observed that preterm infants receiving SSC regularly initiated more positive interactions (e.g., smiling) when reunited with their mothers after a period of separation– a behavior reflecting infants’ involvement with their caregiver. The only study on longitudinal effects of daily SSC on full‐term infants’ behavior to date reported that infants in the intervention condition were more socially bidding toward their mothers at three months of age (Bigelow & Power, 2012). However, this study was not an RCT.

Next to affecting the infant, SSC might also affect the quality of maternal caregiving. Feldman et al. (2014) suggest that close physical contact allows mothers to familiarize with their infants’ cues, enabling them to react more promptly and appropriately. This ability to pick up and interpret infants’ cues is characterized as sensitive caregiving (Ainsworth et al., 1978; Leerkes, 2010). In preterm infants, more sensitive, as well as more affectionate caregiving, has been reported when mothers performed daily SSC (Bigelow et al., 2010; Feldman et al., 2002; Tessier et al., 1998). Additionally, one study in preterm infants reported that mothers performing daily SSC were more cooperative, as they adapted their own actions in order to avoid interference with their infant's autonomous behavior (Feldman et al., 2002). To date, there is a lack of RCTs on the effects of daily SSC on maternal caregiving behavior in infants born full term.

A novel way of assessing dyadic interaction quality is the alignment of mothers’ and infants’ physiological processes— a construct called bio‐behavioral synchrony (DiLorenzo et al., 2021; Feldman, 2012b; Reyna & Pickler, 2009). Synchronization of physiological rhythms emerges in late pregnancy and is suggested to be a critical component of human attachment, shaping later coordination of social behavior (Feldman, 2007; Feldman, 2017). Synchronization of biological processes can aid mother–infant dyads in the regulation of distress (DiLorenzo et al., 2021; Reyna & Pickler, 2009). In the first month after delivery, proximity between mother and infant is suggested to enhance physiological synchrony of the HPA axis. Synchrony of cortisol levels between mothers and preterm infants has, for instance, been found after a period of room sharing at the neonatal intensive care unit (Mörelius et al., 2012). Daily SSC might foster biological mother–infant synchronization in a similar fashion. A study on preterm infants found a correlation of baseline cortisol levels between mothers and infants who had provided SSC, while this correlation was not present in dyads providing CAU (Mörelius et al., 2015). However, no study to date has assessed synchronization of cortisol levels in the presence of a stressor. Additionally, effects of SSC on mother–infant adrenocortical synchrony have not yet been assessed in full‐term infants.

Altogether, the existing body of literature suggests that daily SSC facilitates infants’ stress regulation and improves the interaction quality with their mother. However, there is a lack of RCTs on the potential effects of an SSC intervention for full‐term infants. In the current RCT, we investigated whether daily SSC between mothers and their full‐term infants during the first 5 postnatal weeks improved infants’ stress regulation, by assessing (1a) infants’ cortisol reactions, and (1b) infants’ behavioral reactions, to a mild natural stressor that consisted of the mother bathing the infant and hence included mother–infant interaction throughout the caregiving session. Additionally, we assessed effects of daily SSC on the quality of the mother–infant interaction in terms of (2a) maternal caregiving behavior, and (2b) mother–infant adrenocortical synchrony. We hypothesized that we would find decreased cortisol reactivity, less emotional distress, and increased responsiveness as well as involvement in infants of the SSC condition. We also hypothesized that mothers in the SSC condition would provide more sensitive, cooperative, and affectionate care, and that SSC would facilitate mother–infant adrenocortical synchrony. In order to achieve a comprehensive overview of the data, we additionally explored effects of the intervention on mothers’ cortisol reactivity.

This study is based on secondary outcomes of an RCT (Cooijmans et al., 2017). Previous assessments of this RCT demonstrated positive effects of SSC on the duration of breastfeeding (Cooijmans et al., 2021), as well as on infant crying and sleep (Cooijmans et al., in revision).

## 2 METHODS

### 2.1 Trial design

This RCT assessed two parallel conditions (SSC intervention vs. CAU). The current study focuses on secondary outcomes of this RCT. Primary outcome of this RCT was the effect of SSC on maternal postpartum depression (not addressed in this study). The RCT was reported in accordance with CONSORT guidelines, was registered at the Dutch Trial Register (Trial ID: NL5591), and the study protocol was additionally published (Cooijmans et al., 2017). The ethics committee of the faculty of Social Sciences (Radboud University) approved the trial in 2016 (ECSW2015‐2311‐358).

### 2.2 Participants

**FIGURE 1:**
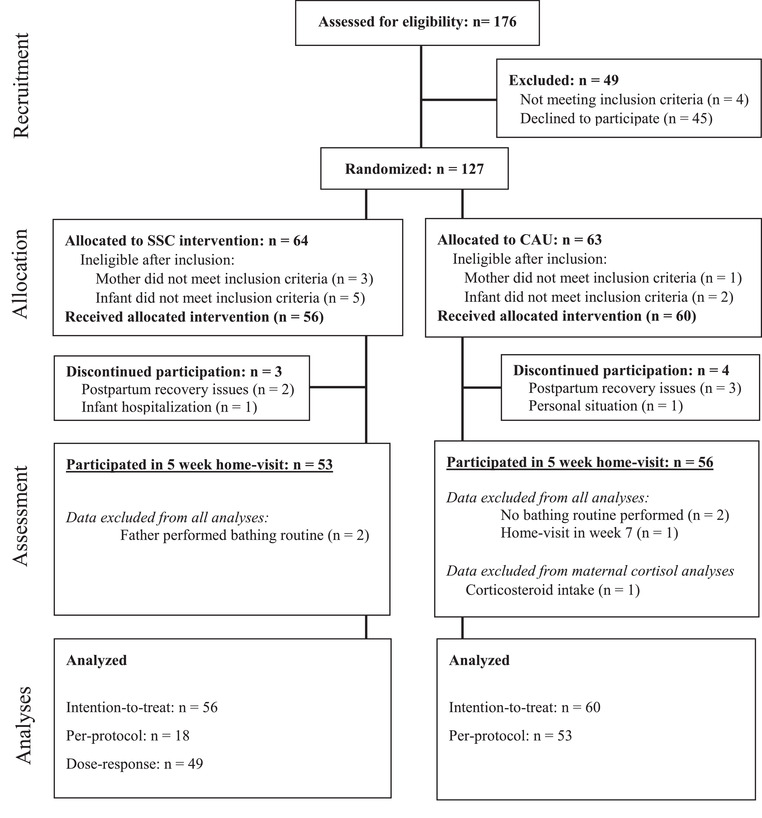
Participant flowchart

Recruitment of 116 pregnant women took place in the region of Nijmegen (the Netherlands) between April 2016 and September 2017. Recruitment took place via social media, flyers, as well as a database of pregnant women interested in participation in scientific studies. Inclusion criteria were: fluency in Dutch, older than 18, no twin pregnancy, no medication or drug use, no serious mental or physical health issues, and no participation in other intervention studies. Inclusion criteria for infants were: born at ≥37 weeks, with a birthweight of at least 2500 g, no congenital anomalies, and a 5‐min APGAR score of 7 or higher. The participant flow is presented in Figure 1.

### 2.3 Randomization and masking

Recruitment included a cover story about the study examining associations between infant feeding behavior, sleep, mother–infant contact, as well as maternal and infant health. Pregnant women were also informed that participation entailed a simple mother–infant contact period after delivery for a subgroup of the sample. With a computer‐generated allocation sequence, mothers were randomly assigned to the SSC or CAU conditions (1:1), with a stratification by parity (primiparae vs. multiparae), using random blocks of four and six. Randomization was performed, and sealed in envelopes, by an independent researcher.

### 2.4 Procedure

#### 2.4.1 Prenatal period

Women were visited by a researcher (K. C.) between weeks 34 and 36 of gestation, received information in line with the cover story, signed informed consent forms, and were allocated to a condition. The SSC condition received detailed verbal and written instructions on the intervention by the researcher, while the CAU condition did not receive further information. Mothers in the SSC condition were instructed to undress the infant, and then position the diapered infant in an upright position on the mother's bare chest (see also Cooijmans et al., 2017). The researcher also provided information regarding optimal positioning and safety during SSC. Then, all women filled in questionnaires on demographics.

#### 2.4.2 Postnatal period

Women in the SSC condition were encouraged to perform one daily uninterrupted hour of SSC from birth until postnatal week 5 (see also Cooijmans et al., 2017). Providing 1 h of SSC uninterruptedly was requested for two reasons. First, a single sleep cycle of a newborn infant lasts approximately 47 min, and hence it would be less likely that infants are woken up during a cycle of sleep if a full hour of SSC is provided (Stern et al., 1969). Second, undressing and dressing an infant has been shown to elicit mild distress, and providing SSC spread over several sessions a day would require infants to be undressed and dressed more often, provoking unnecessary stress in the infant (Jansen et al., 2010b). In both conditions, all mothers were contacted weekly (via telephone, e‐mail, or text message), and reminded to fill in a daily contact logbook. Mothers in the SSC condition were also asked how SSC went and potential obstacles were discussed. None of the mothers reported adverse events or intervention‐related issues.

When the infant was 5 weeks old, a home visit took place during a weekday. As is customary in the Netherlands, all mothers were on maternity leave at this time. In order to take the fluctuation in diurnal cortisol levels in mothers and infants into account, home visits took place between 12 pm and 5 pm. During the visit, mothers bathed their infant according to their usual routine, while being unobtrusively videotaped by the researcher. Bathing produces cortisol increases in infants and has been used as a mild stressor in previous research (Albers et al., 2008; Jansen et al., 2010b; Tollenaar et al., 2011). Saliva was sampled from mother and infant before undressing (baseline, T1), as well as 25 min (poststressor, T2) and 40 min (recovery, T3) after the routine. Infants’ saliva was sampled by gently swabbing the infant's mouth with absorbent eye sponges that were thereafter placed in tubes (de Weerth et al., 2007). Mothers provided saliva in tubes by passive drooling. We investigated cortisol reactivity (T2) as well recovery (T3) from the stressor, to which we refer to as “stress reactivity” throughout the manuscript.

Videotapes were used to score infant behavioral reactions and the quality of maternal caregiving behavior afterward. Mothers also filled in questionnaires on postnatal mental health for the primary aim of this RCT, including questionnaires on postpartum depression (Edinburgh Postnatal Depression Scale; EPDS; Cox et al., 1987) and anxiety (State‐Trait Anxiety Inventory; STAI; Spielberger et al., 1983). During debriefing at infant age one, none of the mothers in the CAU condition reported knowing, nor having heard from others, about the aim of the intervention.

### 2.5 Measures

#### 2.5.1 Skin‐to‐skin contact

Mothers noted periods of contact, including SSC, holding (including breastfeeding), as well as periods of no contact, in a contact logbook for 15‐min time intervals. Mothers kept track of the logbook approximately every 2–3 h, during moments suiting them best throughout the day (i.e., after diaper changes or breastfeeding). Daily durations of SSC were calculated for logbooks when at least 80% of a day was filled in for at least 21 of the 35 days. Additionally, it was assessed on how many days SSC was performed uninterruptedly. Missing days were replaced with the mean amount of SSC of 2 days prior and 2 days after that day for valid logbooks (Beijers et al., 2012). Total SSC during the intervention was calculated in minutes, only for logbooks that contained sufficient data.

#### 2.5.2 Infant stress reactivity

##### Infant cortisol reactions

The eye sponges containing infant saliva were centrifuged and the extracted saliva was stored at −20°C. Cortisol levels were determined at the Laboratory of Endocrinology at UMC Utrecht, with an in‐house competitive radioimmunoassay, by employing a polyclonal anticortisol antibody (K7348), with the tracer [1,2‐3H(N)]‐hydrocortisone (PerkinElmer NET396250UC). The lower detection limit was 1.0 nmol/l, interassay variation was <6% at 2.5–28 nmol/l, and intraassay variation was <4%. Of the available 312 infant samples, 228 samples (CAU: *N* = 116; SSC: *N* = 112) contained sufficient saliva for cortisol determination. Missing samples due to a lack of saliva were evenly distributed over the two groups (CAU: *N* = 43; SSC: *N* = 41).

##### Infant behavioral reactions

Videotapes of the bathing routine were rated by five trained researchers. Infant behavior was rated on responsivity (paying attention and reacting to maternal cues) and involvement (autonomously initiating interactions) on 9‐point scales, and negative mood (showing distress or crying) on a 7‐point scale (Ainsworth et al., 1978). Pearson's correlations (*r*) between scores on the scales Responsivity and Involvement were high (*r* = .90), and the two scales were therefore averaged to a composite score. This strategy of combining variables that assess similar constructs is a common practice, as it reduces the number of outcome variables, and hereby the risk of Type I errors (Song et al., 2013).

Raters double scored 52% of the videos. For double scored videos, the final score was determined by combining the scores of the two raters. If they differed by one point, the score deviating more from the scale mean was chosen, in order to overcome regression toward the mean. If the two scores differed by more than one point, an independent third observer scored the video, and the scores of the two raters agreeing the most on all constructs were chosen for determination of the final scores. Interrater agreement (Weighted Cohen's kappa, *k*) was strong for the ratings of responsivity (*k *= .90), involvement (*k *= .92), and negative mood (*k *= .96).

#### 2.5.3 Interaction quality

##### Quality of maternal caregiving

The videotapes were also rated on maternal caregiving behavior. Mothers were rated on the constructs: sensitivity (*k *= .95; responding appropriately and immediately to infant's cues) and cooperation (*k *= .92; adapting behavior to the needs, and avoiding interference with infant's autonomous behavior) on 9‐point scales, and positive regard (*k *= .82; acting warmly and appreciatively), as well as negative regard (*k *= .81; showing disregard or harshness) on 7‐point scales (Ainsworth et al., 1978). Since the constructs sensitivity and cooperation correlated highly (*r* = .93), an average score was created across the two scales (Song et al., 2013).

##### Mother–infant adrenocortical synchrony

Cortisol levels of mothers’ samples were also determined at the Laboratory of Endocrinology at UMC Utrecht, following the same procedure as infants’ samples. If mothers’ and infants’ cortisol levels had been taken more than 10 min apart, the two samples of that time point were excluded from the synchrony analyses (2 out of the 222 complete mother–infant samples). For synchrony analyses, both mothers’ and infants’ cortisol levels were log transformed in order to achieve a normal distribution.

### 2.6 Statistical approaches

Analyses were performed using R version 3.6.1. (R Core Team, 2021). As in previous studies on this RCT (Cooijmans et al., 2021), the current study was assessed with three different approaches. All mother–infant dyads were included in the intention‐to‐treat approach (ITT), regardless of protocol adherence or withdrawal from the study. In the ITT approach, missing outcome data in the (multivariate) analyses of variance (ANOVAs) were imputed with the expectation‐maximization method (Liu & Brown, 2013). For multilevel model analyses (MLM), no imputation was performed, as MLM is robust for missing data (Snijders & Bosker, 2012). In the per‐protocol approach (PP), dyads of the SSC condition were only included if they had sufficiently filled in the SSC diary (at least 21 of the 35 days), and performed at least 1 h of uninterrupted SSC on ≥28 of the 35 days. Also, in this PP approach, dyads were only included if they had outcome data for the 5‐week assessment, and no data were imputed. In previous studies on this RCT, dyads of both conditions were excluded in the PP approach if they had provided incomplete outcome data (Cooijmans et al., 2021). In this study, dyads with missing cortisol values were not excluded in the PP approach, as missingness was caused by a lack of sufficient saliva for analysis in several infant samples. The exploratory dose–response approach (DR) was performed within the SSC condition, on dyads who had sufficiently filled in the logbook. In the DR analyses, the total duration of SSC in minutes was used as a continuous predictor, and for ANOVAs, imputed data were used. All analyses were repeated excluding dyads with mothers scoring above the clinical cut‐off on the EPDS (score ≥10; Cox et al., 1987) and/or STAI (score ≥40; Spielberger et al., 1983).

### 2.7 Preliminary analyses

Power calculations on the primary outcome (maternal depressive symptoms) retrieved that, taking attrition into account, 116 dyads were required with a power of 80% to detect a medium effect size (*f* = .24) (Cooijmans et al., 2017). For all outcome variables, outliers were identified and winsorized, replacing the score with the mean plus/minus three times the standard deviation (Tukey, 1977). Demographic information and study variables are reported for the ITT and PP samples (Table 1). Group comparisons for continuous variables were assessed with independent sample *t*‐tests if they were normally distributed, and Mann–Whitney *U* tests if they were nonnormally distributed (“stats”; R Core Team, 2021). Group differences on categorical variables were assessed with *χ* ^2^ tests.

**TABLE 1:** Descriptive statistics and group comparisons for mother–infant dyads in the skin‐to‐skin contact (SSC) and care‐as‐usual (CAU) condition

	Intention‐to‐treat			Per‐protocol	
	CAU (*N* = 60)	SSC (*N* = 56)		SSC (*N* = 18)	
	*M* (SD)^a^	*M* (SD)^a^	Statistic	*M* (SD)^a^	Statistic
Baseline characteristics					
Maternal age (years)	32.48 (3.05)	32.36 (3.85)	1611.00^b^	32.90 (3.80)	524.00^b^
Maternal educational level	6.87 (1.79)	6.82 (1.55)	1495.50^b^	6.78 (1.48)	463.50^b^
Smoking (% No)	100.00	96.43	2.18^c^	96.43	3.38^c^
Alcohol (% No)	100.00	98.21	1.08^c^	98.21	3.38^c^
C‐section (% No)	32.48 (3.05)	32.36 (3.85)	1611.00^b^	32.90 (3.80)	524.00^b^
APGAR score	9.70 (.62)	9.84 (.42)	1499.50^b^	9.72 (.58)	534.50^b^
Infant sex (% girls)	43.33	58.93	2.82^c^	58.93	1.76^c^
Weight at birth (grams)	3567.47 (385.77)	3650.05 (414.93)	−1.11^d^	3760.56 (4.56)	−1.79^d^
Gest. age at birth (weeks)	40.02 (1.10)	40.08 (1.01)	1648.50^b^	40.16 (1.03)	501.50^b^
Age at home visit (days)	39.98 (2.66)	40.56 (3.79)	1235.00^d^	40.61 (2.55)	404.50^d^
Birth order (%)					
First	46.70	48.22	.03^c^	48.21	1.83^c^
Second	38.30	32.14		32.12	
Third	15.00	19.64		19.64	
Total duration SSC (min.)	308.17 (442.41)	2067.68 (850.65)	−11.95^***c^	2905.90 (497.52)	−19.99^***c^
Covariates^[Link]^, ^[Link]^					
Total duration bath (sec.)	820.38 (180.25)	826.96 (197.01)	1188.00^b^	840.41 (247.06)	438.50^d^
Bath position (% sitting up)	24.53	17.65	.45^c^	11.11	.82^c^
Infant stress reactivity^e^					
Infant cortisol (nmol/L)					
Baseline	10.53 (6.35)	9.90 (3.59)	−.24^d^	9.39 (3.15)	.17^d^
Poststressor	14.55 (8.49)	12.82 (5.63)	.27^d^	14.19 (6.47)	−.34^d^
Recovery	11.74 (5.04)	11.59 (4.29)	−.16^d^	13.64 (4.50)	−1.17^d^
Infant behavior					
Responsivity–involvement	4.53 (1.48)	4.41 (1.43)	1731.50^b^	4.11 (1.42)	550.00^b^
Negative mood	3.85 (1.88)	3.40 (1.94)	1894.00^b^	3.56 (2.03)	509.50^b^
Interaction quality^e^					
Mat. caregiving quality					
Sensitivity–cooperation	5.95 (2.07)	6.19 (1.84)	1576.50^b^	5.86 (1.64)	501.50^b^
Positive regard	4.92 (1.51)	5.24 (1.30)	1486.50^b^	4.89 (.90)	485.00^b^
Negative regard	1.25 (.51)	1.24 (.55)	1718.00^b^	1.44 (.78)	422.00^b^
Cort. difference scores^g^					
Baseline	4.99 (5.56)	3.56 (3.27)	.88^d^	3.19 (2.74)	.75 ^d^
Poststressor	8.16 (8.57)	6.68 (5.16)	.62^d^	8.02 (5.99)	−.12^d^
Recovery	6.08 (5.37)	5.52 (3.79)	.73^d^	7.83 (3.73)	−1.15^d^
Maternal cortisol (nmol/L)					
Baseline	7.24 (2.08)	6.98 (2.48)	.89^d^	7.08 (2.97)	.61^d^
Poststressor	7.07 (1.78)	7.36 (2.44)	−.39^d^	7.07 (1.92)	.02^d^
Recovery	6.65 (1.74)	6.95 (2.15)	−.50^d^	6.57 (1.74)	.20^d^

*Notes*. *M*, mean; SD, standard deviation; Gest., gestational; Mat, maternal.

^a^ *M* and SD are presented as nonimputed and nontransformed data.

^b^Mann–Whitney *U* tests for nonnormally distributed data.

^c^ *χ* ^2^ tests for categorical data.

^d^ Independent samples *t*‐tests for data normally distributed after square root transform.

^e^Winsorized data are presented for all moderator and outcome variables with outliers.

^f^Standardized data are presented for all moderators.

^g^Absolute values of maternal minus infant cortisol.

*p < .05; ^**^ *p* < .01; ^***^ *p *< .001.

### 2.8 Main analyses

#### 2.8.1 Infant stress reactivity

##### Infant cortisol reactions

To examine whether SSC had an effect on infants’ adrenocortical stress reactivity, multilevel growth curve models (MLM) were performed on infants’ log transformed cortisol levels (“*lme4*”; Bates et al., 2015). Linear time (exact sample timing in minutes) and intercept were added as random effects, and linear, as well as quadratic time were added as fixed effects. Covariates were added in a build‐up fashion if they led to a decrease of the Watanabe‐Akaike Information Criterion (WAIC; Hamaker et al., 2011). Potential covariates were bathing duration, and position during the bathing routine (horizontal: bathtub vs. vertical: tummy tub), since these variables differed based on maternal choice. Condition (total amount of SSC in DR approach) was entered as a fixed effect. Interactions of condition with time were only added if the WAIC decreased (Hamaker et al., 2011).

##### Infant behavioral reactions

Effects of condition (total amount of SSC in DR approach) on infant behavior during the stressor were assessed with multivariate analyses of variance (MANOVA; R Core Team, 2021). Dependent variables were infant negative mood, and the composite score of responsivity and involvement. Since the assumption of a multivariate normal distribution was not met, the dependent variables were square root transformed.

#### 2.8.2 Interaction quality

##### Quality of maternal caregiving

Condition effects on the quality of maternal caregiving behavior were assessed with a MANOVA including the composite of maternal Sensitivity and Cooperation, as well as positive and negative regard (“stats”; R Core Team, 2021). Dependent variables were square root transformed.

##### Mother–infant adrenocortical synchrony

Two MLM models were used in order to assess group differences in mother–infant synchrony in terms of (1) maternal cortisol predicting infant cortisol, and (2) mother–infant synchrony across baseline, poststressor, and recovery (Nofech‐Mozes et al., 2019). In the first MLM, infant cortisol was predicted with the interaction between condition and maternal cortisol. Linear time was added as a random effect, and linear as well as quadratic time, potential moderators, maternal cortisol, condition, and the interaction of condition with maternal cortisol were added as fixed effects. The three‐way interaction between maternal cortisol, condition and time could not be assessed in this analysis due to a lack of power. A second MLM was performed in order to assess mother–infant adrenocortical synchrony over time. In this MLM, absolute values of difference scores (maternal minus infant cortisol) were predicted by condition. The interaction of condition with time was added based on the WAIC (Hamaker et al., 2011).

An additional MLM was performed on the effect of condition on mothers’ cortisol reactivity, including linear sampling time and intercept as random effects, and linear, as well as quadratic time, potential covariates, and condition as fixed effects.

## 3 RESULTS

### 3.1 Missing data and outliers

Cortisol concentrations for infant analyses were missing for 28 samples at baseline, 23 samples at poststressor, and 23 samples at recovery due to a lack of saliva. Overall, 27% of the infant cortisol concentrations were missing in the SSC condition, and 21% were missing in the CAU condition. Maternal cortisol levels were missing for one sample of the SSC condition at baseline, and no maternal samples were missing at poststressor and recovery. One mother of the CAU condition was excluded due to corticosteroid intake. Five outliers on infant cortisol, and two outliers on maternal cortisol were winsorized. There were no outliers on other outcome variables. Two videotapes were missing for analyses on infant behavior and maternal caregiving quality due to technical problems (e.g., recording inadvertently stopped after a few minutes).

### 3.2 Preliminary analyses

Group comparisons of demographic information and outcome variables are displayed in Table 1. Mothers in the CAU condition provided 308 min (SD = 442), and mothers in the SSC condition provided 2068 min (*SD* = 851) of SSC throughout the intervention phase. In the SSC condition, 18 mothers provided sufficient SSC for the PP approach (>60 consecutive minutes on at least 80% of the days); these mothers provided 2906 min (SD = 498) of SSC. Mann–Whitney *U* tests showed that the mean daily duration of SSC performed was significantly higher in the SSC condition than in the CAU condition for the ITT and the PP approach (Table 1). The clinical cut‐off on the EPDS was reached by five mothers in the CAU and five mothers in the SSC condition. On the STAI, seven mothers in the CAU condition and five mothers in the SSC condition scored above the clinical cut‐off. Sensitivity analyses excluding these mothers from the analyses indicated no change in the results.

### 3.3 Main analyses

#### 3.3.1 Infant stress reactivity

##### Infant cortisol reactions

Table 2 displays outcomes of the MLM on infant cortisol reactivity. There were no significant differences between conditions in infant cortisol. The effect of quadratic time was significant in the ITT, PP, and DR approaches. Infants’ cortisol levels increased at poststressor and decreased again at recovery.

**TABLE 2:** Multilevel growth curve models for intention‐to‐treat, per‐protocol, and dose–response approaches.

	Intention‐to‐treat		Per‐protocol		Dose–response	
Infant cortisol^[Link]^, ^[Link]^	*B* (SE)	*t*	*B* (SE)	*t*	*B* (SE)	*t*
Infant cortisol^[Link]^, ^[Link]^						
Intercept	2.462 (.062)	39.78^***^	2.485 (.073)	33.87^***^	2.464 (.059)	41.88^***^
Linear time	.001 (.002)	.47	.002 (.003)	.62	.022 (.044)	.49
Quadratic time	−.001 (.000)	−4.30^***^	−.001 (.000)	−3.92^***^	−.120 (.040)	−2.98^**^
Duration	−.088 (.039)	−2.24^*^	/	/	/	/
Condition	−.008 (.077)	−.11	−.015 (.118)	−.12	−.003 (.042)	−.06
Mother–infant adrenocortical synchrony^[Link]^, ^[Link]^						
Outcome: infant cort.						
Intercept	2.879 (.350)	8.22^***^	3.019 (.390)	7.73^***^	2.173 (.233)	9.31^***^
Linear time	.001 (.002)	.36	.001 (.003)	.50	.018 (.044)	.40
Quadratic time	−.001 (.000)	−4.20^***^	−.001 (.000)	−3.96^***^	−.116 (.040)	−2.90^**^
Duration	−.100 (.040)	−2.50^*^	/	/	/	/
Condition	−.829 (.441)	−1.88^†^	−1.144 (.601)	−1.90^†^	.078 (.242)	.32
Mat. cortisol	−.222 (.179)	−1.24	−.283 (.199)	−1.42	.151 (.117)	1.29
Condition × Mat. cort.	.435 (.229)	1.91^†^	.596 (.311)	1.92^†^	−.042 (.122)	−.35
Outcome: diff. score^c^						
Intercept	.725 (.055)	13.23^***^	.722 (.064)	11.28^***^	.654 (.055)	11.83^***^
Linear time	.003 (.002)	1.68	.005 (.002)	2.13^*^	.041 (.039)	1.05
Quadratic time	−.000 (.000)	−2.67^**^	−.000 (.000)	−1.79	−.106 (.038)	−2.78^**^
Duration	−.059 (.033)	−1.76	/	/	−.103 (.043)	−2.38^*^
Condition	−.081 (.064)	−1.27	−.044 (.094)	−.46	.034 (.042)	.80
Maternal cortisol^[Link]^, ^[Link]^						
Intercept	1.917 (.040)	48.19^***^	1.914 (.038)	49.74^***^	1.946 (.048)	40.28^***^
Linear time	−.002 (.001)	−1.98^*^	−.002 (.001)	−2.48^*^	−.002 (.020)	−.12
Quadratic time	−.000 (.000)	−3.10^**^	−.000 (.000)	−2.24^*^	−.042 (.016)	−2.68^**^
Duration	.070 (.028)	2.54^*^	.089 (.031)	2.87^**^	−.071 (.047)	1.52
Condition	−.005 (.055)	−.09	−.010 (.070)	−.14	−.003 (.046)	−.07

*Notes*. SE, standard error ; mat., maternal; cort, cortisol.

^a^Cortisol values were log transformed.

^b^Duration of skin‐to‐skin contact as a continuous predictor for dose–response analyses.

^c^Absolute value of maternal–infant cortisol.

^†^ *p* < .10, ^*^ *p* < .05, ^**^ *p* < .01, ^***^ *p *< .001.

##### Infant behavioral reactions

Results of the MANOVAs on ratings of infants’ behavior during the bathing routine did not reveal significant differences between conditions in the ITT, PP, or DR approaches (Table 3).

**TABLE 3:** Multivariate analysis of variance on effects of condition on square‐root transformed infant behavioral reactions and quality of maternal caregiving for intention‐to‐treat, per‐protocol, and dose–response approaches

	Intention‐to‐treat				Per‐protocol				Dose–response			
	*Λ*	*η* ^2^	*F*(1,114)	*p*	*Λ*	*η* ^2^	*F*(1,68)	* p *	*Λ*	*η* ^2^	*F*(1,47)	*p*
Infant behavior												
Condition^a^	.962	.040	2.225	.113	.935	.066	2.300	.103	.980	.020	.461	.634
Maternal caregiving												
Conditio^a^	.983	.017	.642	.590	.976	.024	.540	.655	.917	.083	1.357	.268

*Note. Λ*, Wilks’ Lambda. *η* **^2^,** eta squared.

^a^Duration of skin‐to‐skin contact as a continuous predictor for dose–response analyses.

#### 3.3.2 Interaction quality

##### Quality of maternal caregiving

MANOVAs on maternal caregiving behavior during the bathing routine did not show an effect of condition in the ITT, PP, or DR approaches (Table 3).

##### Mother–infant adrenocortical synchrony

**FIGURE 2:**
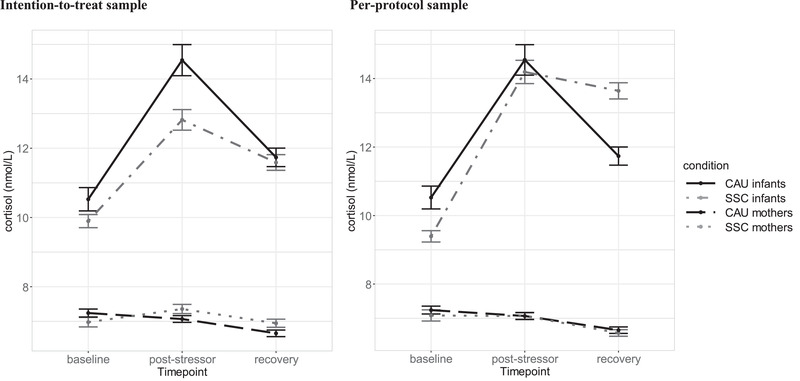
Cortisol levels of infants and mothers at baseline, poststressor (25 min), and recovery (40 min) for the skin‐to‐skin contact (SSC) and care‐as‐usual (CAU) condition in the intention‐to‐treat (left) and the per‐protocol (right) approaches

Outcomes of both analyses regarding mother–infant synchrony are displayed in Table 2. In the first MLM, the interaction of maternal cortisol levels with condition on infant cortisol was marginally significant in the ITT (*b* = .435, SE = .229, *t* = 1.91, *p* = .057) and PP (*b* = ‐.601, SE = .311, *t* = 1.93, *p* = .055) approaches. Compared with the CAU condition, cortisol of mothers in the SSC condition were overall more similar to infants’ cortisol levels (Figure 2). MLM on mother–infant cortisol difference scores did not show significant effects of condition.

There were no significant differences of maternal cortisol reactivity between conditions (Table 2).

## 4 DISCUSSION

The aims of this study were to assess whether full‐term infants receiving a daily SSC intervention, compared with CAU, in the first 5 postnatal weeks showed (1) lower stress reactivity, in terms of cortisol and behavioral reactivity, and (2) improved mother–infant interaction quality, in terms of maternal caregiving quality and mother–infant adrenocortical synchrony, during a bathing routine provided by the mother. Contrary to our hypotheses, we did not find significant effects of daily SSC on infants’ stress reactivity and mother–infant interaction quality in the ITT, PP, and DR analyses. Interestingly, we did find marginally significant effects in ITT and PP analyses indicating that maternal cortisol concentrations tended to be more alike to infants’ cortisol concentrations in the SSC group as compared with the CAU group.

Potentially, the SSC intervention revealed no significant effects on our outcomes due to the low compliance with the protocol of mothers in the SSC condition. Despite elaborate instructions and regular contact with participating mothers, not all mothers provided one uninterrupted hour daily. Summing up all SSC performed a day, about one third of the SSC mothers regularly performed one hour of SSC. Similarly, a previous study on full‐term infants also reported relatively low protocol compliance (Hardin et al., 2020). This study only found facilitating effects of SSC on infant cortisol reactivity in their PP analyses, including a small sample of infants who had actually received the recommended hour of SSC (Hardin et al., 2020). In the current assessment, we performed similar PP analyses. However, since only 18 mothers were included in these analyses, the PP analyses were underpowered. The other study on the effects of SSC on full‐term infants reported higher intervention compliance, and found beneficial effects of SSC on infants’ behavioral stress regulation (Bigelow & Power, 2012). Mothers in this previous study, however, were informed about the aims of the intervention beforehand which may have induced a sampling bias, as mothers might have only signed up if they were interested in SSC. Contrary to the previous study, we used a cover story and randomly allocated mothers to the conditions irrespective of their interest in SSC, which presumably led to lower intervention compliance.

It is also possible that SSC effects did not become apparent due to the timing and nature of the assessment. For instance, SSC effects on infants’ stress regulation and the mother–infant interaction quality might emerge at a later age. A study in full‐term infants showed that mothers’ quality of caregiving behavior was not associated with infants’ cortisol reactivity to a bathing session at five weeks of age (Jansen et al., 2010a), while another study revealed that higher quality of maternal caregiving behavior was related to decreased cortisol reactivity to a bathing situation at three months of age (Albers et al., 2008). In addition, the current assessment focused on direct effects of daily SSC on the infant HPA axis reactivity, which is part of the sympathetic nervous system. A review of a family interventions including SSC in neonatal intensive care units, however, indicated that repeated mother–infant contact sessions facilitate infants’ development of the ability to regulate parasympathetic states (Porges et al., 2019; Welch et al., 2017). Finally, while the current study assessed infant and maternal behavior separately, future studies might explore potential effects of daily SSC on the autonomous emotional connection between the dyad, as a facilitator of infants’ biobehavioral stress regulation (Hane et al., 2019; Porges et al., 2019; Welch et al., 2017).

The absence of effects might also be explained due to the nature of the stressor. SSC may not have an effect on reactions to mild physical stressors, such as a bathing session, but it might have an effect on infant stress reactions to other types of stressors (e.g., socioemotional and novel stressors; Puhakka & Peltola, 2020), or infants’ stress levels throughout the day. A previous experimental study on full‐term infants found that one SSC episode decreased infants’ stress levels immediately, but that subsequent cortisol reactions to a bathing session were increased (Beijers et al., 2016). In addition, while the current study found no evidence that SSC was associated with infant behavioral stress reactivity to a bathing session, the same RCT revealed in another study that SSC was associated with decreased daily crying and fussing during the first 12 postnatal weeks (Cooijmans et al., in revision). Future studies should investigate whether daily SSC also decreases infants’ cortisol concentrations throughout the day, ideally after the circadian rhythm has matured in the second half of the first year of life (de Weerth et al., 2003).

The last explanation for the absence of significant SSC effects might be that SSC does not affect full‐term infants as much as it affects infants born preterm or with low birth weight. To our knowledge, all RCTs demonstrating benefits of SSC for infants’ stress reactivity and mother–infant interaction quality were performed with infants born preterm or with a very low weight (Mörelius et al., 2015; Tessier et al., 1998). While full‐term infants are usually cared for in proximity immediately after birth (e.g., carried, held, breastfed), preterm infants are more vulnerable as their neurodevelopment is strained (Fleiss & Gressens, 2019; Norholt, 2020) and they additionally experience less physical contact (i.e., due to incubator care). Potentially, a subgroup of full‐term infants and/or mothers might have benefitted from the SSC intervention, such as dyads exposed to adversity and risks. For instance, full‐term infants who are exposed to maternal stress during pregnancy showed altered stress reactivity (Tollenaar et al., 2011), and these infants and their mothers might have benefitted from SSC, but this hypothesis remains for future research.

The current study has substantial strengths. We used a RCT with blind recruitment, and the drop‐out rate was low throughout the intervention phase. However, the current study also suffered limitations: many infant samples lacked sufficient saliva for analysis, producing missing data. Even though MLM is robust for missing data (Snijders & Bosker, 2012), the lack of power did not allow us to look at more complex three‐way interactions, or more elaborate time‐lagged synchrony effects. Finally, our study did not include a diverse sample in terms of ethnicity, socio‐economic status, or maternal age, making it less representative of the population.

## 5 CONCLUSION

The current RCT did not find evidence of effects of daily SSC on infant stress reactivity or the quality of the mother–infant interaction. Further research should assess whether daily SSC affects full‐term infants’ daily levels of distress. Additionally, future studies are required to explore possibilities to enhance adherence to the intervention and unveil other potential underlying mechanisms of SSC effects in full‐term infants.

## CONFLICT OF INTEREST

The authors do not have conflicts of interest to declare.

## Data Availability

The research data are part of an ongoing study, but can be requested from the first author via e‐mail for scientific purposes.
